# Prevalence of potential drug‒drug interactions and associated factors among elderly patients in Ethiopia: a systematic review and meta-analysis

**DOI:** 10.1186/s41256-024-00386-7

**Published:** 2024-11-13

**Authors:** Tekletsadik Tekleslassie Alemayehu, Yilkal Abebaw Wassie, Abaynesh Fentahun Bekalu, Addisu Afrassa Tegegne, Wondim Ayenew, Gebresilassie Tadesse, Demis Getachew, Abebaw Setegn Yazie, Bisrat Birke Teketelew, Mekonnen Derese Mekete, Setegn Fentahun, Tesfaye Birhanu Abebe, Tefera Minwagaw, Gebremariam Wulie Geremew

**Affiliations:** 1 Department of Social and Administrative Pharmacy, School of Pharmacy, College of Medicine and Health Sciences, University of Gondar, Gondar, Ethiopia; 2 Department of Clinical Pharmacy, School of Pharmacy, College of Medicine and Health Sciences, University of Gondar, Gondar, Ethiopia; 3 Department of Pharmacology, School of Pharmacy, College of Medicine and Health Sciences, University of Gondar, Gondar, Ethiopia; 4 Department of Pharmaceutical Chemistry, School of Pharmacy, College of Medicine and Health Sciences, University of Gondar, Gondar, Ethiopia; 5 Department of Medical Nursing, School of Nursing, College of Medicine and Health Sciences, University of Gondar, Gondar, Ethiopia; 6 Department of Hematology and Immune Hematology, School of Laboratory, College of Medicine and Health Sciences, University of Gondar, Gondar, Ethiopia; 7 Department of Psychiatry, School of Medicine, College of Medicine and Health Sciences, University of Gondar, Gondar, Ethiopia; 8 Department of Internal Medicine, School of Medicines College of Medicine and Health Sciences, University of Gondar, Gondar, Ethiopia; 9 Department of Medical Parasitology, School of Biomedical and Laboratory Sciences College of Medicine and Health Sciences, University of Gondar, Gondar, Ethiopia; 10 Department of Pharmacy, Bahir Dar University, Bahir Dar, Ethiopia; 11 Department of Pharmacy, Debremarkos University, Debremarkos, Ethiopia

**Keywords:** Prevalence, Potential drug‒drug interaction, Associated factors, Elderly, Ethiopia

## Abstract

**Background:**

The occurrence of potential drug‒drug interactions (pDDIs) is a serious global issue that affects all age groups, with the elderly population being the most vulnerable. This is due to their relatively high rates of comorbidity and polypharmacy, as well as physiological changes that can increase the potential for DDIs and the likelihood of adverse drug reactions. The aim of this study was to estimate the prevalence of pDDIs and associated factors among elderly patients in Ethiopia.

**Methods:**

A comprehensive literature search using the preferred reporting items for systematic review and meta-analysis statement was conducted on HINARI, Science Direct, Embase, PubMed/MEDLINE, Google Scholar, and Research Gate. Data were extracted via a Microsoft Excel spreadsheet and analyzed via STATA version 11.0. Egger regression tests and funnel plot analysis were used to check publication bias, and the I^2^ statistic was used to evaluate statistical heterogeneity. Sensitivity and subgroup analyses were also conducted to identify potential causes of heterogeneity.

**Results:**

Seven articles were analyzed, and a total of 1897 pDDIs were identified in 970 patients, resulting in an average of 1.97 DDIs per patient. The number of DDIs per patient ranged from 0.18 to 5.86. The overall prevalence of pDDIs among elderly patients was 50.69% (95% CI 18.77–82.63%). However, the prevalence of pDDIs ranged widely from 2.80 to 90.1%. When the severity of the interactions was considered, the prevalence of potential DDIs was found to be 28.74%, 70.68%, and 34.20% for major, moderate, and minor pDDIs, respectively. Polypharmacy and long hospital stays were identified as factors associated with pDDIs among elderly patients in Ethiopia.

**Conclusions:**

The overall prevalence of pDDIs among elderly patients was high, with a wide range of prevalence rates. Moderate-severity interactions were the most prevalent. Polypharmacy and long hospital stays were identified as factors associated with pDDIs among elderly patients. The study suggests that DDIs identification database itself could have modified the DDIs prevalence rate. As a result, a single DDIs identification database needs to be authorized; otherwise, clinical knowledge should be taken into account when interpreting the information obtained.

**Supplementary Information:**

The online version contains supplementary material available at 10.1186/s41256-024-00386-7.

## Background

Drug‒drug interactions (DDIs) are the most common type of interaction in the medical field. They are defined as a “*pharmacological or clinical response to the administration of a combination of drugs that differs from the expected effects of the individual drugs when taken alone*. *This can also refer to a quantitative change in the toxicity or effectiveness of a medication when it is taken with another drug*” [1]. DDIs can be classified as actual DDIs or potential drug‒drug interactions (pDDIs). Actual DDIs are identified from patient adverse outcomes; however, pDDIs are those identified through analysis of the pharmacologic profiles of each drug used by patients and identification of possible adverse events due to the association [2]. Not all pDDIs result in an adverse outcome; therefore, the occurrence of actual DDIs is lower than that of pDDIs [3]. DDIs can also be classified on the basis of their severity and the mechanism how they interact. They can range from mild to severe and can be categorized as pharmacokinetic (PK), pharmacodynamics (PD) or mixed interaction [4, 5].

The occurrence of DDIs is a serious global issue for patient safety, affecting individuals of all age groups. However, older adults aged 60 years and above are particularly vulnerable [6]. Despite this vulnerability, clinical trials are often conducted on younger adults, which can make it challenging to provide appropriate care for the elderly population [7]. Owing to the numerous health issues and physiological changes associated with aging, older patients often require more medications than younger patients do [8], increasing their susceptibility to DDIs [9]. This is because physiological changes associated with aging can affect the PKs and PDs of drugs, potentially increasing the risk of drug toxicity and adverse drug reactions (10). As a result, DDIs are frequently unavoidable in this population, and elderly individuals are particularly vulnerable to the adverse outcomes of these interactions [11].

According to a systematic analysis of the literature, the pooled prevalence of pDDIs globally was 28.8%. However, the prevalence rate ranged widely from 8.34% to 100%, with the number of DDIs per 100 patients ranging from 120 to 3060 [12]. The high prevalence of pDDIs in elderly patients can be attributed to several factors, such as the age of the patient, the number of comorbidities and medications, the pharmacokinetic and pharmacodynamics nature of the drugs, and the influence of disease on drug metabolism [8, 11, 13]. The types of drugs, such as cardiovascular drugs, which are commonly involved in DDIs, also contribute to the high prevalence of pDDIs [8, 12, 14].

Likewise, the occurrence of pDDIs among elderly patients is also common in different African countries, with rates ranging from 23 to 84.8% [15–17]. In addition to global associated factors, long hospital stays, hypertension, diabetes mellitus, and prescriber issues, such as multiple drug prescriptions by multiple prescribers, inadequate knowledge of prescribers on DDIs, and poor recognition of the relevance of DDIs by prescribers, have been strongly linked to the occurrence of DDIs in Africa [16–18]. Like in other African countries, pDDIs are a national issue in Ethiopia. A systematic literature review in Ethiopia revealed that the national prevalence of pDDIs among the general population was 72.2%. Similarly, polypharmacy, age of the patients, comorbid diseases, and long hospital stays were also the risk factors associated with pDDIs in Ethiopia [19].

Importantly, DDIs can have both desirable and undesirable effects [4], and they are a significant cause of adverse drug reactions (ADRs) and hospital admissions. In fact, the incidence of DDI-related ADRs in older adults has been estimated to range from 4.5 to 6.5% [20, 21]. In elderly patients, clinically significant DDIs can also lead to deterioration of overall health, decreased quality of life, longer hospital stays, increased need for ambulatory services, and higher healthcare costs [22–24]. Furthermore, a systematic review revealed that pDDIs were responsible for 4.8% of hospital admissions in elderly patients, compared with only 0.57% in the general population [25]. Some DDIs may not immediately cause noticeable changes in patients but can still result in treatment failure [26]. Despite the implementation of automated DDIs alert systems, which have helped decrease the occurrence of DDIs, DDIs remain an evolving public health problem [27]. However, the numerous alerts produced by these systems can lead to alert fatigue among physicians and pharmacists, resulting in a significant number of overrides of DDIs alerts [28]. As a result, DDIs continue to pose a serious risk to public health.

Given the growing elderly population and the potential impact of DDIs, to date, no systematic review has explicitly addressed the prevalence of pDDIs and associated factors among elderly patients in Ethiopia. This study provides a comprehensive understanding of the nature and extent of DDIs prevalence and associated factors in this growing and vulnerable population in Ethiopia. Therefore, the aim of this study was to estimate the pooled prevalence of pDDIs and associated factors among elderly patients in Ethiopia.

## Method

### Study protocol

The protocol for this systematic review and meta-analysis is registered in the international prospective registration of systemic reviews (PROSPERO) with the ID CRD42024524838. The current review employed a PRISMA (Preferred Reporting Items for Systematic Reviews and Meta-Analyses) compliant methodology for searching the literature, selecting studies, extracting data, and reporting conclusions [29].

### Search strategy

A systematic review and meta-analysis were conducted to determine the prevalence of pDDIs and associated factors among elderly patients in Ethiopia. The search for relevant research articles was conducted via databases such as HINARI, Science Direct, Embase, Thesis Bank, PubMed/MEDLINE, Google Scholar, and Research Gate for English-language publications. The reference lists of the identified studies were also reviewed for additional relevant research. The search was conducted between May 19, 2024, and June 10, 2024, and all published articles available online until the day of data collection were considered. The search terms used included "prevalence," "occurrence," "pharmacoepidemiology," "potential DDIs," "drug‒drug interactions," "inappropriate medication use," "associated factors," "predictors," "elderly," "elder," "older adults," "aged," and "Ethiopia." These terms were used in combination with "AND" and "OR" to identify relevant articles. After data were retrieved from the articles, we attempted to contact the primary or corresponding authors via email to obtain any missing information.

### Study selection

After relevant articles were identified through database searches, duplicate publications were removed, and the articles were imported into the EndNote program X9 version. Three investigators, TTA, GWG, and YAW, worked independently during this phase. Next, the three investigators screened the titles and abstracts to determine the eligibility of the articles. Finally, using the inclusion criteria outlined below, the same investigators independently assessed the full-text articles. Any disagreements were resolved through discussion before the further analysis was conducted.

### Eligibility criteria

#### Inclusion criteria

Studies are included based on: a) observational studies (cohort, cross-sectional, and case‒control studies); b) being reported as original articles, theses, and abstracts from scientific events and meetings; c) being published in English at any time; and d) addressing the prevalence of pDDIs among adults aged 60 years and above with any disease and admitted to hospital wards or visiting outpatient clinics.

#### Exclusion criteria

Based on the consensus of the authors, we decided to exclude articles: (a) not reporting the prevalence of pDDIs and/or associated factors but only characterizing DDIs in the population of interest; (b) reporting interventions for pDDIs but not providing their prevalence before the intervention; (c) analyzing the prevalence of DDIs in adults, including elderly individuals without providing enough data to calculate the prevalence of DDIs in the elderly population of the study, either in the original document or after the information was requested; and (d) not published in peer-reviewed journals. We also excluded pilot, qualitative, validation, unpublished reports, and seminar presentations. Additionally, studies with incomplete data, even after the authors were contacted, were also excluded. If there were doubts about the eligibility of a study, the decision was made by involving three additional reviewers (AAT, AFB, SF and WA).

### Data extraction

Data were extracted and managed in a predesigned form in Microsoft Excel. Following the selection of the articles and the final decisions, TTA, AAT, YAW, GWG and TM separately extracted all relevant data from the articles. The authors entered the following data in a standard data extraction form: the first author's name, publication year, countries in which the study was conducted, study design, pathology, target population, study setting, interaction database, number of patients, number of patients with DDIs, and lists of medication classes that caused the interactions. Additionally, the outcome of interest (prevalence of pDDIs—major, moderate and minor) and associated factors of pDDIs, as well as measures of effect (odds ratios (ORs)), lower confidence intervals, and upper confidence intervals, were also extracted. In cases where the authors had different opinions during the data extraction process, they discussed it until they reached an agreement. At this point, the data were double-extracted with other authors. To compare the observed and expected agreements across authors, we used kappa statistics to illustrate any differences. To determine the reliability of the meta-analytic results, a sensitivity analysis was also performed.

### Outcome measurements

The primary aim of the systematic review and meta-analysis was to assess the pooled prevalence of pDDIs, which was calculated as the percentage of patients who presented with at least one DDI among the total number of patients studied, as well as the factors associated with pDDIs. This study also included three secondary outcomes: (i) to characterize pDDIs on the basis of their severity (major, moderate, and minor) and mechanism of action (PK, PD and mixed interactions); (ii) to determine the number of DDIs per patient, defined as the number of DDIs divided by the number of patients with at least one pDDI; and (iii) to identify the most common drug class involved in pDDIs.

### Quality assessment

Owing to the cross-sectional nature of the studies included, study quality was assessed via the Agency for Healthcare Research and Quality (AHRQ) methodological checklist for cross-sectional and prevalence studies [30]. This assessment tool consists of an 11-item questionnaire that evaluates the quality of data collection, inclusion criteria, outcome measurement, and other measurements. The items were answered as yes ( +), no (–) or unclear (U) for the study. TBA, GN, SF, G, and D conducted the quality assessment. Any disagreements between reviewers were resolved through consensus, and the opinion of another reviewer (WA, AS, or BBT) was sought if necessary. The quality assessment process was completed on June 20, 2024.

### Statistical procedures

After the data were extracted and imported into Microsoft Excel, STATA 11.0 was used for analysis. The outcomes of the primary articles were presented via text, tables, and forest plots. For each original article, we calculated the standard error of prevalence via the binomial distribution. Furthermore, to determine whether there was publication bias in the included articles, two methods were employed. A funnel plot was used to visualize the symmetric distribution and the absence of publication bias in the included articles. Egger's correlation and Begg's regression intercept tests were employed at the 5% significance level. In the event that our analysis revealed publication bias, we utilized funnel plots, estimated the number and outcome of missing articles, and accounted for the hypothetically absent articles via the nonparametric "trim and fill" approach developed by Duval and Tweedie.

### Heterogeneity assessment

Der Simonian and Laird's pooled effects of pDDIs were estimated via a random effects meta-analysis approach. Heterogeneity between articles was assessed by considering the I^2^ inconsistency statistic. Significant levels of heterogeneity were considered present when the I^2^ estimate was greater than or equal to 70%. Additionally, if we found evidence of heterogeneity during analysis, we used a sensitivity analysis to pinpoint its potential cause. We applied a leave-one-out sensitivity analysis to determine the potential cause of heterogeneity in the pooled prevalence of pDDIs. This was accomplished by systematically eliminating one author or one article at a time.

### Subgroup analyses

The prevalence of pDDIs among elderly patients was examined by subgrouping the countries where the study was conducted, drug-drug interaction databases, study designs, pathologies, and study settings. The prevalence of pDDIs is reported as percentages with 95% confidence intervals (CIs).

## Results

### Article search results

**Fig. 1:**
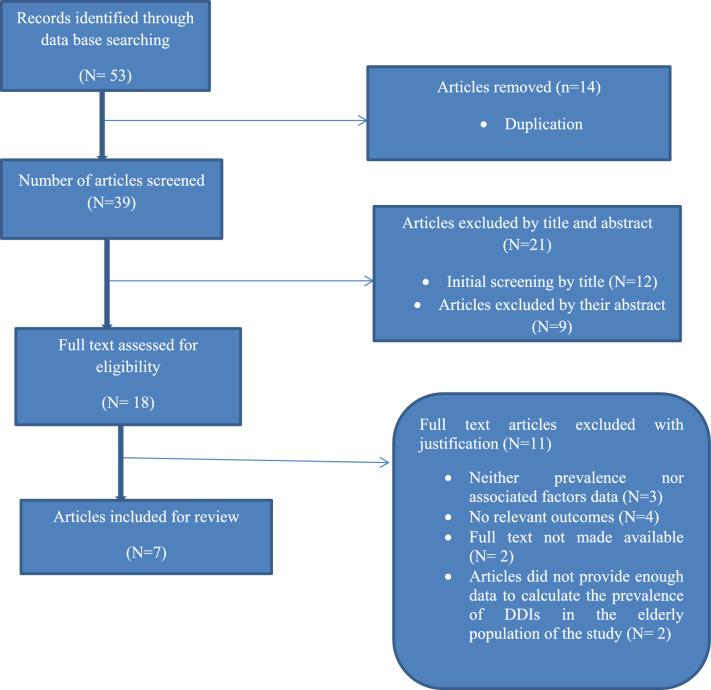
PRISMA flowchart diagram

A total of 53 articles were identified from the databases. After removing duplicates, 39 articles remained for screening. Of these, 21 articles were excluded based on their titles and abstracts. The remaining 18 articles were then assessed according to predetermined inclusion criteria. After this assessment, 11 articles were excluded. Ultimately, 7 full-text articles that met the eligibility criteria and passed the quality assessment were included in the final systematic review and meta-analysis (Fig. 1).

### General characteristics of the included studies

Seven primary articles, comprising 2251 individuals, were included in the final systematic review and meta-analysis on the prevalence of pDDIs and associated factors among elderly patients. Among the seven articles, one focused solely on pDDIs. All of the articles utilized observational cross-sectional study designs, with three being retrospective and two being prospective. The designs of the remaining two studies were not specified. The included articles were published between 2014 and 2022. Geographically, the articles were obtained from two regions and one city administration (Addis Ababa). The articles included patients with various types of diseases, both in medical wards and outpatient settings. Five articles analyzed patients with all types of pathologies, whereas two focused specifically on patients with cardiovascular disorders. Three articles investigated DDIs in inpatient wards, three in outpatient settings, and one in both settings. Five different DDIs identification databases were used to detect pDDIs, with only one article utilizing two DDIs identification databases. Medscape online DDIs database systems were used in three articles (42.9%), Micromedex® was used in two articles (28.6%), and the Beers criteria were used in the remaining two articles (28.6%). (Table 1 for details.)

**Table 1:** Characteristics of the studies included in this systematic review and meta-analysis on PDDI among elderly patients in Ethiopia

Authors	Publication year	Study design	Region	Database	Sample size	Pathology	Prevalence of pDDIs (%)
Teni et al. [36]	2014	Retrospective cross-sectional	Amhara	Micromedex	392	All	24.9
Teka et al. [34]	2016	Cross-sectional	Tigri	Micromedex	140	All	62.2
Assefa et al. [35]	2020	Retrospective cross-sectional	Addis Ababa	Medscape online	168	CVD	86.31
Bhagavathula et al. [32]	2021	Hospital based cross-sectional	All region	Beers criteria	320	All	2.8
Adem et al. [31]	2022	Retrospective cross-sectional	Addis Ababa	AGS & MAI	384	CVD	90.1
Dagnew et al. [33]	2022	Prospective observational	Amhara	Medscape online	389	All	58.1
Dagnew et al. [37]	2022	Prospective observational	Amhara	Medscape online	389	All	30.62

### Quality of the included studies

Four studies [31–34] were rated as being of high methodological quality, and the remaining three studies [35–37] were judged to have moderate methodological quality. A full description of the AHRQ methodological quality assessment and ratings for each of the seven studies included in this systematic review and meta-analysis is provided in (Supplementary Material 2).

### Study outcome measures

#### Pooled prevalence of pDDIs among elderly patients in Ethiopia

**Fig. 2:**
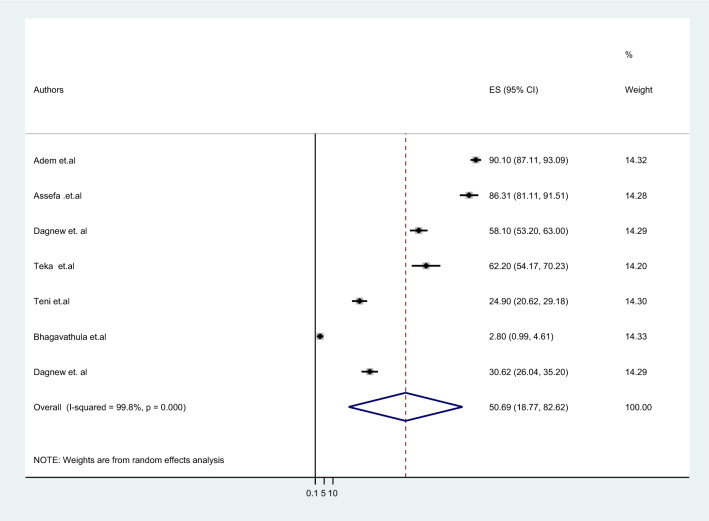
The pooled prevalence of pDDIs among elderly patients in Ethiopia

The results revealed that the pooled prevalence of pDDIs among elderly patients in Ethiopia was 50.69% (95% CI 18.77–82.63%) (Fig. 2). The included articles reported a wide range of pDDIs prevalence rates among elderly patients in Ethiopia, from 2.8 [32] to 90.1% [31]. When the severity of the interactions was considered, the pooled prevalence of potential DDIs was found to be 28.74%, 70.68%, and 34.2% for major, moderate, and minor pDDIs, respectively (Table 5). Only one article classified the prevalence of pDDIs among elderly patients on the basis of the mechanism of the interactions, reporting 21.29%, 73.06%, and 5.65% for PK, PD, and mixed DDIs, respectively [35]. A total of 1897 pDDIs were identified in 970 patients, resulting in an average of 1.97 DDIs per patient. The number of DDIs per patient ranged from 0.18 to 5.86.

### Factors associated with the prevalence of pDDIs among elderly patients in Ethiopia

Polypharmacy and long hospital stays were factors associated with pDDIs among elderly patients in Ethiopia (Fig. 4).

### Common interacting drug classes

The current systematic review revealed that the classes of drugs that interacted the most often were cardiovascular drugs [31–35, 37], gastrointestinal drugs [31–35, 37], anti-infective drugs [32–35, 37] and endocrine drugs [32–35, 37].

### Test of heterogeneity and publication bias, subgroups and sensitivity analysis

#### Heterogeneity and publication bias

**Fig. 3:**
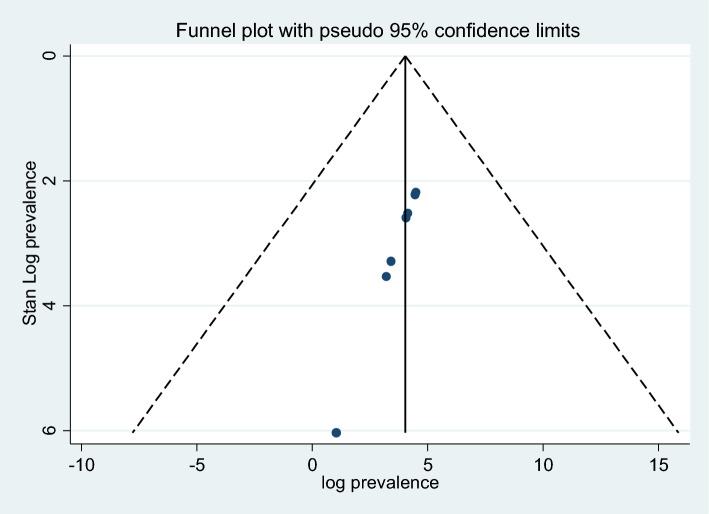
Random effects funnel plot of logit event rate of pDDIs effect sizes by standard error

The heterogeneity of the seven articles included in the current systematic review and meta-analysis was high, as shown by the test statistics (I^2^ = 99.8%, *p* value = 0.000). A funnel plot was used to demonstrate the symmetrical distribution and lack of publication bias in the included papers (Fig. 3). Additionally, *p* = 0.180 indicates that Egger’s test was used to verify that there was no publication bias (Table 2).

**Table 2:** Egger’s test of the PDDI among elderly patients in Ethiopia

Std_Eff	Coef	Std. Err	t	*P* >|t|	[95% Conf. Interval]
Slope	− 8.572581	29.85 897	− 0.29	0.786	− 85.32749 68.18233
Bias	26.92122	17.33927	1.56	0.180	− 17.59079 71.55324

### Subgroup analysis

The current review revealed that there were differences in the prevalence of pDDIs depending on the DDIs identification database, region where the articles were conducted, study design, pathology, and study setting. Subgroup analysis by region revealed that the highest prevalence of pDDIs was observed in Addis Ababa, at 88.83% (95% CI 85.32–93.09%), followed by Amhara, with a prevalence of 37.85% (95% CI 18.31–57.38%). Furthermore, subgroup analysis based on the DDIs identification database revealed that the highest prevalence of pDDIs was detected by Medscape online, at 58.32% (95% CI 26.82–89.83%), followed by the Micromedex DDIs database, at 43.39% (95% CI 6.84–79.94%). With respect to the study design employed, the highest prevalence of pDDIs was observed in studies that utilized a retrospective cross-sectional study design, at 67.11% (95% CI 25.21–109.01%). Moreover, subgroup analysis was also performed based on the clinical diagnosis of the patients, and the highest prevalence was found in studies that assessed the pDDIs among CVD patients, at 88.83% (95% CI 85.32–92.34%), compared with 35.59% (95% CI 12.34–58.84%) in studies that assessed the pDDIs among all clinical conditions. Finally, in the study setting, the prevalence of pDDIs was greater in inpatient settings, at 50.17% (95% CI 29.46–70.87%), than in outpatient settings, at 39.26% (95% CI −18.30–96.83%) (Table 3).

**Table 3:** Subgroup analysis of the pDDIs among elderly patients in Ethiopia

Variable	Subgroup	Number of studies	Prevalence/% (95% CI)	I^2^%	*p* value
Region	Amhara	3	37.85 (18.31, 57.38)	98.2	0.000
	Addis Ababa	2	88.83 (85.32, 93.09)	34.9	0.215
	Other	2	32.37 (− 25.84, 90.58)	99.5	0.000
Study design	Retrospective cross- sectional	3	67.11 (25.21, 109.01)	99.7	0.000
	Prospective observational	2	44.35 (17.42, 71.28)	98.4	0.000
	Cross-sectional	2	32.37 (− 25.84, 90.58)	99.5	0.000
Database	Medscape online	3	58.32 (26.82, 89.83)	99.2	0.000
	Micromedex	2	43.39 (6.84, 79.94)	98.5	0.000
	Other	2	46.44 (− 39.11, 131.99)	100	0.000
Pathology	CVDs	2	88.83 (85.32, 92.34)	34.9	0.215
	All conditions	5	35.59 (12.34, 58.84)	99.4	0.000
Setting	Outpatients	3	39.26 (− 18.30, 96.83)	99.9	0.000
	Inpatients	3	50.17 (29.46, 70.87)	97.6	0.000
	Both	1	86.31 (81.11, 91.51)	–	–

Other: Beers criteria & Medication Appropriate Index (MAI)

### Sensitivity analysis

**Fig. 4:**
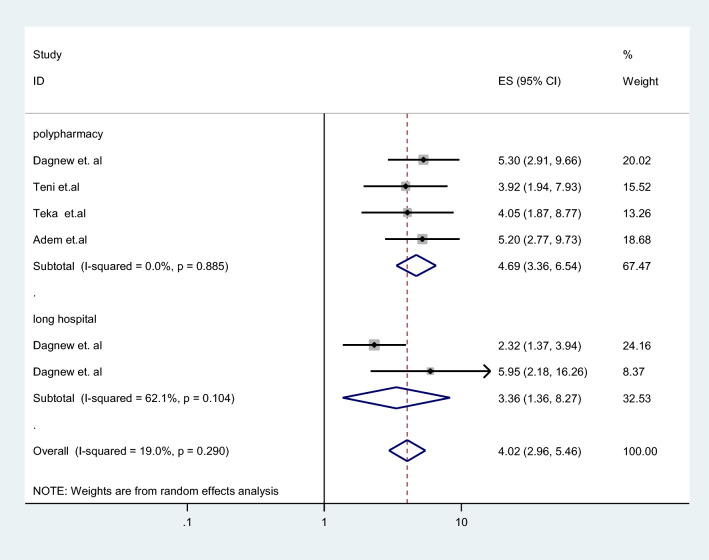
pooled factors associated with pDDIs among elderly patients in Ethiopia

Sensitivity analysis was performed in the current systematic review and meta-analysis to investigate the impact of each study on the pooled prevalence of pDDIs among elderly patients (Fig. 4). The fact that all of the numbers fall within the anticipated 95% CIs suggests that the omission of one study did not significantly change the prevalence in this review. Furthermore, there was no significant change in the degree of heterogeneity even if an attempt was done to eliminate one or more of the studies from the analysis. Therefore, seven studies were included for the final systematic review and meta-analysis (Table 4).

**Table 4:** Sensitivity analysis of the PDDI among elderly patients in Ethiopia

Authors	Estimate prevalence (95% CI)	Heterogeneity	
		I2 (%)	*p* value
Adem et al.	44.09 (16.76, 71.41)	99.6	0.000
Assefa.et al.	44.76 (10.89, 78.63)	99.8	0.00
Dagnew et al.	49.46 (13.32, 85.61)	99.8	0.000
Teka et al.	48.79 (13.79, 83.79)	99.8	0.000
Teni et al.	55 (17.83, 92.48)	99.8	0.000
Bhagavathula et al.	58.70 (33.76, 83.64)	99.4	0.000
Dagnew et al.	54.05 (16.90, 91.19)	99.8	0.000

## Discussion

The objective of the current systematic review was to estimate and offer a quantitative summary of the prevalence of pDDIs, as well as associated factors, among elderly patients in Ethiopia. The analysis included seven articles with a total of 2251 individuals. The overall pooled prevalence of pDDIs among elderly patients in Ethiopia was 50.69% (95% CI 18.77–82.63%). This is consistent with the global pooled prevalence of pDDIs among elderly patients (28.8%) [38] and studies conducted on the general population in Ethiopia (72.2%) [19]. This may be due to similar healthcare practices, prescribing patterns, and the common use of certain medications across different populations. Standard treatment guidelines for various diseases often recommend similar classes of medications, resulting in similar risks of DDIs for patients with the same diseases.

In terms of the severity of DDIs, the prevalence rates of major and moderate DDIs were 28.74% and 70.68%, respectively. This finding is in line with other studies that reported similar outcomes. A systematic review across the globe reported pooled prevalence of major and moderate DDIs of 28.9% and 54.4%, respectively [39]. This consistency may be due to similarities in patient demographics, methodologies, and criteria used to identify and classify DDIs. However, the prevalence of pDDIs in the current study was higher than that reported in previously conducted studies in Albania (0.8%) [40], Australia (15%) [41], and the USA (7.7%, 10.4%) [42, 43]. This may be due to socioeconomic factors, such as education levels, healthcare infrastructure, and public health initiatives, which can influence how medications are prescribed and managed. These factors may lead to differences in how drug interactions are handled in different countries. Additionally, differences in clinical conditions, study settings, and criteria used to identify and classify DDIs may also contribute to these discrepancies. In contrast, the findings of the current systematic review were lower than those of a study conducted in Croatia (90.6%) [44]. This may be attributed to the presence and effectiveness of pharmacovigilance systems, which monitor and address adverse drug reactions and interactions. These systems may be more robust in Ethiopia, leading to a lower prevalence of pDDIs. Access to healthcare and medication can also differ, with Ethiopia potentially having limited access to certain drugs, making it easier to recognize and avoid pDDIs. In contrast, Croatia, with potentially better access to a wider range of medications, may have a greater risk of pDDIs. In addition to these factors, differences in healthcare systems and practices, such as prescription practices and the monitoring and management of drug interactions, can also influence the occurrence of pDDIs. Compared with Croatia, Ethiopia may have stricter guidelines or better monitoring systems, leading to fewer interactions.

The wide variation in prevalence estimates for pDDIs identified by this systematic review is similar to that reported in recent reviews. For example, one review reported that prevalence estimates for pDDIs among elderly patients ranged from 0.8 to 90.6% [38], whereas another reported a range of 8.34–100% [12]. The wide variation in the prevalence of pDDIs in the current systematic review and meta-analysis may be due to differences in clinical conditions, the number of comorbidities and medications, and DDIs identification databases used to identify pDDIs. Previous research also supports this explanation [45]. The high prevalence of pDDIs reported by some studies may be attributed to prescriber issues such as multiple drug prescriptions by multiple prescribers, inadequate knowledge of prescribers on pDDIs, or poor recognition of the relevance of pDDIs [18]. Additionally, certain types of drugs, such as cardiovascular medications, which are commonly involved in pDDIs [8, 12, 14], may contribute to the wide variation in prevalence estimates of this systematic review, as most articles included in this study measure the prevalence of pDDIs for cardiovascular medications. Therefore, studies reporting a high prevalence of pDDIs should be acknowledged.

However, when the prevalence estimates were pooled in a meta-analysis, there was significant heterogeneity between studies, which could be explained by differences in the DDIs identification databases used to identify pDDIs, regions, study settings, and study designs. Subgroup analyses based on the DDIs identification databases used showed wide variation in pooled prevalence estimates, ranging from 43.39 to 58.32%. This finding is consistent with a recent review that also reported differences in prevalence estimates on the basis of the DDIs identification database used [38]. While several DDIs screening software programs are available, one limitation is their lack of clinical relevance, which can result in the over reporting of pDDIs [46]. Additionally, the information obtained from one DDIs identification database may differ from that of another. This means that the software itself may have influenced the prevalence estimates. Ideally, multiple sources should be used, and the information should be interpreted carefully. Micromedex® is considered the gold standard and a generic measurement [47]. However, in this review, only two studies assessed pDDIs with Micromedex®, and one study evaluated pDDIs via more than one database.

Furthermore, the subgroup analysis revealed that the prevalence of pDDIs differed based on the study setting, with a higher prevalence in inpatient settings than in outpatient settings, which revealed that the prevalence of pDDIs was high in inpatient settings (50.17% versus 39.26% in the outpatient setting). This may be due to the use of specialized treatments and medications in inpatient settings, which may have unique interaction profiles. Additionally, the inpatient setting often has more complex and severe conditions, leading to frequent adjustments in medication, which can result in unanticipated interactions. The severity of illness may also necessitate the use of high-risk medications, which have greater potential for interactions [48].

Polypharmacy and long hospital stays were significantly associated with pDDIs. Polypharmacy is a major risk factor for pDDIs. This finding is in line with a systematic review conducted on the general population in Ethiopia, which revealed that taking five or more medications is an independent factor that leads to pDDIs [19]. The current findings are also in line with those of cross-sectional studies conducted in Iran, Brazil and India, which indicated that taking six or more medications is an important factor for the occurrence of pDDIs [49–51]. This may be attributed to each additional drug increasing the likelihood of interactions. This is supported by a study from Brazil, which revealed that as the number of medications taken by a patient increased, so did the probability of pDDIs [52]. Elderly patients may also require polypharmacy because of their comorbidities. Managing multiple medications can be challenging and increase the risk of medication errors. Long hospital stays, particularly more than seven days, were also associated with the occurrence of pDDIs, which is consistent with previous research [19, 53]. Hospitalized patients are more likely to have multiple illnesses, comorbid conditions, and chronic therapeutic regimens, as well as frequent changes in their medication regimens, which can increase the risk of pDDIs [54].

The current systematic review and meta-analysis highlights the need to adapt standardized methods to identify DDIs to narrow the wide range of prevalence across studies. The DDIs identification database itself could have modified the prevalence of pDDIs. Hence, the use of DDIs databases with different sensitivities can overestimate and underestimate the prevalence rate of pDDIs. Therefore, a single DDIs identification database needs to be authorized; otherwise, a national list of DDIs, which is regularly updated to reflect both current clinical practice and emerging evidence of clinically important DDIs, needs to be developed and maintained. This encourages consistency in reporting the prevalence of DDIs and reduces the amount of alerts fatigue among health professionals. Furthermore, the pooled prevalence of pDDIs was high. These findings suggest that elderly patients are a natural high-risk population for pDDIs. DDIs are also frequently unavoidable and often predictable medical issues after patients experience adverse outcomes. As a result, each patient should be evaluated individually, pDDIs should be characterized, the risk–benefit ratio should be weighed, and prompt interventions should be implemented to improve the quality of care for the elderly population. Finally, drugs used to treat cardiovascular disorders are frequently prescribed to elderly individuals to treat conditions associated with aging and are involved in the majority of drug‒drug interactions. Therefore, healthcare providers in geriatric cardiovascular treatment facilities should prioritize screening, monitoring, and providing special attention to elderly patients (Table 5).

**Table 5:** Pooled prevalence of pDDIs by type among elderly patients in Ethiopia

Types of DDIs	Number of study	Prevalence (95% CI)	Heterogeneity	
			I2 (%)	*p*- value
Major DDIs	4	28.74 (5.27, 52.21)	98.9	0.000
Moderate DDIs	5	70.68 (55.60, 85.76)	98.1	0.000
Minor DDIs	5	34.20% (− 1.02, 69.42)	99.6	0.000
PK DDIs	1	21.29	–	–
PD DDIs	1	73.06	–	–
Mixed (PK& PD) DDIs	1	5.65	–	–

### Limitations

The pooled effect of pDDIs among elderly patients has several limitations. First, the articles included in the current review focused only on pDDIs and did not address actual DDIs because of a lack of studies. This means that the evaluation of pDDIs may overestimate the actual DDI rates, as not every pDDI leads to an actual DDI. Second, there was heterogeneity among the included articles, which may have affected the results of this systematic review and meta-analysis. For example, the study revealed that prevalence estimates differed by region. Addis Ababa had the highest prevalence of pDDIs, followed by the Tigri and Amhara regions. Unfortunately, the review could not include other regions, as no articles from these regions were included in the study. It remains to be invesitigated whether there are significant differences in the prevalence of pDDIs among elderly patients across other regions. Therefore, caution should be taken when interpreting the findings. Fourth, the severity of pDDIs may have been defined differently in each article, as several different methods were utilized to identify pDDIs.

## Conclusions

The current systematic review and meta-analysis revealed a high prevalence of pDDIs among elderly patients in Ethiopia, and the most prevalent DDIs were moderate according to severity. However, there was significant heterogeneity between studies, which could be explained by differences in the DDIs identification databases used to identify pDDIs, regions, clinical conditions, study settings, and study designs. Factors such as long hospital stays and polypharmacy were associated with pDDIs among elderly patients in Ethiopia. The study suggests that the DDIs identification database itself could have modified the DDIs prevalence rate. As a result, a single DDIs identification database needs to be authorized; otherwise, clinical knowledge should be taken into account when interpreting the information obtained.

## Supplementary Information

Supplementary Material 1: PRISMA checklist for pDDI.

Supplementary Material 2: Quality scores.

Supplementary Material 3: Sample data extraction format.

## Abbreviations

ADR: Adverse drug reactions
AGS: American Geriatrics Scale
CI: Confidence interval
CVDs: Cardiovascular diseases
DDIs: Drug‒drug interactions
MAI: Medication appropriate index
pDDIs: Potential drug‒drug interactions
PKs: Pharmacokinetic
PDs: Pharmacodynamics

## Declarations

### Availability of data and materials

All relevant data are available within the manuscript.
