# Correction: Prevalence of potential drug‒drug interactions and associated factors among elderly patients in Ethiopia: a systematic review and meta-analysis

**DOI:** 10.1186/s41256-024-00402-w

**Published:** 2024-12-30

**Authors:** Tekletsadik Tekleslassie Alemayehu, Yilkal Abebaw Wassie, Abaynesh Fentahun Bekalu, Addisu Afrassa Tegegne, Wondim Ayenew, Gebresilassie Tadesse, Demis Getachew, Abebaw Setegn Yazie, Bisrat Birke Teketelew, Mekonnen Derese Mekete, Setegn Fentahun, Tesfaye Birhanu Abebe, Tefera Minwagaw, Gebremariam Wulie Geremew

**Affiliations:** 1 Department of Social and Administrative Pharmacy, School of Pharmacy, College of Medicine and Health Sciences, University of Gondar, Gondar, Ethiopia; 2 Department of Clinical Pharmacy, School of Pharmacy, College of Medicine and Health Sciences, University of Gondar, Gondar, Ethiopia; 3 Department of Pharmacology, School of Pharmacy, College of Medicine and Health Sciences, University of Gondar, Gondar, Ethiopia; 4 Department of Pharmaceutical Chemistry, School of Pharmacy, College of Medicine and Health Sciences, University of Gondar, Gondar, Ethiopia; 5 Department of Medical Nursing, School of Nursing, College of Medicine and Health Sciences, University of Gondar, Gondar, Ethiopia; 6 Department of Hematology and Immune Hematology, School of Laboratory, College of Medicine and Health Sciences, University of Gondar, Gondar, Ethiopia; 7 Department of Psychiatry, School of Medicine, College of Medicine and Health Sciences, University of Gondar, Gondar, Ethiopia; 8 Department of Internal Medicine, School of Medicines College of Medicine and Health Sciences, University of Gondar, Gondar, Ethiopia; 9 Department of Medical Parasitology, School of Biomedical and Laboratory Sciences College of Medicine and Health Sciences, University of Gondar, Gondar, Ethiopia; 10 Department of Pharmacy, Bahir Dar University, Bahir Dar, Ethiopia; 11 Department of Pharmacy, Debremarkos University, Debremarkos, Ethiopia

**Correction: Global Health Research and Policy (2024) 9:46** 10.1186/s41256-024-00386-7

Following publication of the original article [1], the authors reported an error in the given name of the fifth author, it needed to be updated from “Wendim Ayenew” to “Wondim Ayenew”.

The correct author name has been provided in this Correction.

The original article [1] has been corrected.
