# Supplementary material for: Prevalence of potential drug‒drug interactions and associated factors among elderly patients in Ethiopia: a systematic review and meta-analysis
Source: Glob Health Res Policy. 2024 Nov 13;9:46. doi: 10.1186/s41256-024-00386-7 (PMC11559191; doi:10.1186/s41256-024-00386-7)
Supplement: Supplementary file 2 — Supplementary Material 2: Quality scores. [file 41256_2024_386_MOESM2_ESM.docx]

Table_S2 Quality assessment of potential drug-drug interaction and its associated factors for the included studies in this systematic review and meta-analysis.

| Author, year of  (Publication) | Q1 | Q2 | Q3 | Q4 | Q5 | Q6 | Q7 | Q8 | Q9 | Q10 | Q11 | Total score |
| --- | --- | --- | --- | --- | --- | --- | --- | --- | --- | --- | --- | --- |
| Adem et.al (2022) | + | + | + | + | U | + | + | + | U | + | U | 9.5 |
| Assefa.et.al (2020) | + | U | + | U | + | + | U | - | U | + | U | 7.5 |
| Dagnew et. al (2022) | + | + | + | + | U | + | + | + | + | + | - | 9.5 |
| Teka et.al (2016) | + | + | + | + | U | + | + | + | + | + | U | 10 |
| Teni et.al (2014) | + | + | + | + | U | + | - | U | U | + | - | 7.5 |
| Bhagavathula et.al (2021) | + | + | + | U | - | + | + | + | + | + | U | 9 |
| Dagnew et.al (2022) | + | + | + | U | + | + | U | + | - | + | - | 8 |

**Key:** Each item use "Yes (+= 1 point)", "Unclear (U =0.5 point)", or "No (- =0 point)"

**Question codes:**

1. Define the source of information (survey, record review)
2. List inclusion and exclusion criteria for exposed and unexposed subjects (cases and controls) or refer to previous publications
3. Indicate time period used for identifying patients
4. Indicate whether or not subjects were consecutive if not population-based
5. Indicate if evaluators of subjective components of study were masked to other aspects of the status of the participants
6. Describe any assessments undertaken for quality assurance purposes (e.g., test/retest of primary outcome measurements)
7. Explain any patient exclusions from analysis
8. Describe how confounding was assessed and/or controlled
9. If applicable, explain how missing data were handled in the analysis
10. Summarize patient response rates and completeness of data collection
11. Clarify what follow-up, if any, was expected and the percentage of patients for which incomplete data or follow-up was obtained
